# Cost-effectiveness analysis of a text message system for COVID-19 testing for K-12 school communities

**DOI:** 10.1093/jpids/piaf111

**Published:** 2026-01-22

**Authors:** Maura Dougherty, Yelena P Wu, Leighann Kolp, Hannah L Brady, Tammy K Stump, Tatyana V Kuzmenko, Minkyoung Yoo, Jonathan Chipman, Guilherme Del Fiol, Kimberly A Kaphingst, Adam L Hersh, Kelly J Lundberg, Brian Orleans, Jennifer Wirth, David W Wetter, Richard E Nelson

**Affiliations:** Department of Economics, University of Utah, 260 Central Campus Drive,Gardner Commons, Room 4100, Salt Lake City, UT 84112, United States; Department of Dermatology, University of Utah, 30 N. Mario Capecchi Dr., Salt Lake City, UT 84112, United States; Huntsman Cancer Institute, University of Utah, 2000 Circle of Hope Drive, Salt Lake City, UT 84112, United States; Huntsman Cancer Institute, University of Utah, 2000 Circle of Hope Drive, Salt Lake City, UT 84112, United States; Huntsman Cancer Institute, University of Utah, 2000 Circle of Hope Drive, Salt Lake City, UT 84112, United States; Department of Dermatology, University of Utah, 30 N. Mario Capecchi Dr., Salt Lake City, UT 84112, United States; Huntsman Cancer Institute, University of Utah, 2000 Circle of Hope Drive, Salt Lake City, UT 84112, United States; Department of Biomedical Informatics, University of Utah, 421 Wakara Way #140, Salt Lake City, UT 84108, United States; Division of Epidemiology, University of Utah, 295 Chipeta Way Salt Lake City, UT 84132, United States; Huntsman Cancer Institute, University of Utah, 2000 Circle of Hope Drive, Salt Lake City, UT 84112, United States; Department of Population Health Sciences, University of Utah, 295 Chipeta Way, Williams Building, Room 1N410, Salt Lake City, UT 84108, United States; Department of Biomedical Informatics, University of Utah, 421 Wakara Way #140, Salt Lake City, UT 84108, United States; Huntsman Cancer Institute, University of Utah, 2000 Circle of Hope Drive, Salt Lake City, UT 84112, United States; Department of Communication, University of Utah, 255 Central Campus Dr, Salt Lake City, UT 84112, United States; Department of Pediatrics, University of Utah, 295 Chipeta Way, Salt Lake City, UT 84108, United States; Department of Psychiatry, University of Utah, 501 Chipeta Way, Salt Lake City, UT 84108, United States; Huntsman Cancer Institute, University of Utah, 2000 Circle of Hope Drive, Salt Lake City, UT 84112, United States; Department of Population Health Sciences, University of Utah, 295 Chipeta Way, Williams Building, Room 1N410, Salt Lake City, UT 84108, United States; Huntsman Cancer Institute, University of Utah, 2000 Circle of Hope Drive, Salt Lake City, UT 84112, United States; Huntsman Cancer Institute, University of Utah, 2000 Circle of Hope Drive, Salt Lake City, UT 84112, United States; Department of Population Health Sciences, University of Utah, 295 Chipeta Way, Williams Building, Room 1N410, Salt Lake City, UT 84108, United States; Division of Epidemiology, University of Utah, 295 Chipeta Way Salt Lake City, UT 84132, United States; IDEAS Center, George E. Whalen Department of Veterans Affairs Healthcare System, 500 Foothill Drive Bldg. 182 Salt Lake City, UT 84148, United States

**Keywords:** COVID-19, cost-effectiveness analysis, school health services, mobile health, infectious disease

## Abstract

**Background:**

During the COVID-19 pandemic, school closures led to loss of school-based resources and substantial learning losses for children. To facilitate the return to in-person learning, schools across the US partnered with health agencies to implement strategies such as on-site and at-home COVID-19 testing programs. We aimed to quantify the cost-effectiveness of SCALE-UP Counts, a project that used text messaging and health navigation interventions to promote equitable COVID-19 testing among K-12 school students and their families.

**Methods:**

Families of children from sixteen K-12 schools in Utah were randomly assigned to one of three intervention arms from 2022-2023: unidirectional text messages regarding availability of free COVID-19 test kits [UC], intensive bidirectional text messaging with testing guidance and ability to request test kits [ITM], and intensive bidirectional text messaging plus health navigation [ITM + HN]. Expected cost and effectiveness of each approach was measured. Effectiveness was measured as missed school days avoided, missed workdays avoided, and COVID-19 tests taken, and calculated as ratios of differences over differences in costs. The analysis was performed using a decision analytic simulation model with probabilistic sensitivity analysis.

**Results:**

ITM + HN yielded most missed school days avoided (8290 vs. 1840) and COVID-19 tests taken (9468 vs. 1876) but was costlier than UC ($34 vs. $11 per family). The costs for ITM + HN compared to UC were $30/COVID-19 test taken, and $21/missed workday avoided. ITM alone did not yield improved outcomes relative to UC or ITM + HN.

**Conclusions:**

Inclusion of a health navigator substantially enhances the benefits of bidirectional text messaging compared to UC but is costlier. This study quantifies these extra costs to inform decision makers as to the optimal screening and communication strategy for a school population during a pandemic.

## INTRODUCTION

By August 2023, the COVID-19 pandemic had led to over 6 million hospitalizations and 1 million deaths in the US^1^ with widespread impact on work, community and school activities.^2-4^ COVID-19 case counts for US school-aged children exceeded 15 million by May 2023.^5^ Individuals from historically marginalized populations and families of low socioeconomic status were disproportionately affected.^6-10^ In addition to greater incidence of hospitalization and severe infection,^6^^,^^7^ school absenteeism rates were higher among Black and Hispanic/Latino students compared to non-Hispanic White students.^8^ To slow COVID-19 transmission and implement disease prevention measures, almost all K-12 schools in the US were closed to in-person instruction beginning March 2020.^4^ Over 50 million children were affected by school closures and the transition to home-based learning, which included loss of school-based services such as free/reduced meals, health care, and mental health services.^11^^,^^12^ Students also experienced substantial learning losses.^7^^,^^8^

A priority for school officials was to return to in-person instruction while mitigating the spread of COVID-19 and maintaining student and staff safety. The Centers for Disease Control and Prevention (CDC) recommended developing protocols for providing school-based surveillance (symptom screening) as well as symptomatic (diagnostic) testing as part of school re-opening plans.^13^ Schools across the country partnered with state and local health agencies to employ a variety of strategies, including onsite and at-home testing to facilitate transitions to in-person learning.^14-17^ Testing programs successfully decreased infection rates and missed school days, including among low-income and other vulnerable populations.^14^ However, questions of whether programs provided adequate support to maximize engagement and consistency with testing protocols led school districts to seek programs that provided additional resources and support to students, their families, and school staff.^14-17^ Further, existing programs to promote testing suggested lack of uptake, despite the importance of testing to control mortality and morbidity from the virus.^18^^,^^19^

To address these needs, we initiated a project, SCALE-UP Counts, which tested the effectiveness of population-based interventions (i.e., text messaging and health navigation) to promote equitable COVID-19 testing among K-12 school students and their families, especially among underserved communities. The project sought to increase access to COVID-19 testing and guidance, with the goal of minimizing disruptions to in-person learning. The parents/guardians of students received access to free at-home COVID-19 test kits and were randomly assigned to receive (i) usual care (UC, unidirectional messaging on test access through school or direct mail); (ii) intensive bidirectional text messages (ITM) with testing guidance and ability to request COVID-19 test kits; or (iii) ITM plus the opportunity to speak to a health navigator about COVID-19 testing (ITM + HN). Text messaging was chosen as a low-cost intervention tool that has been used in preventive health interventions in many populations, especially historically disadvantaged groups.^20^^,^^21^ Health navigation has been shown in studies to decrease disparities by addressing barriers and concerns to accessing health care services.^22^^,^^23^ The current analysis examined the cost-effectiveness of the SCALE-UP Counts strategies to increase access to COVID-19 testing and reduce school days and workdays missed for school communities. While the COVID-19 pandemic no longer currently poses an emergency, the results of the current study could inform interventions to address future emergencies and other infectious diseases of public health concern.

## METHODS

### Study Sample or Population

The study received a waiver of documentation of consent from the University of Utah Institutional Review Board allowing for expedited recruitment and enrollment (IRB_00143340). The current analysis included data collected from parents/guardians of students from 16 schools in Utah from 2022-2023. Parents were eligible if their child attended a participating school and the parent had a mobile phone that receives calls and text messages. See Supplemental Material for detailed description of the SCALE-UP Counts study.

### Model Overview

**Figure 1:**
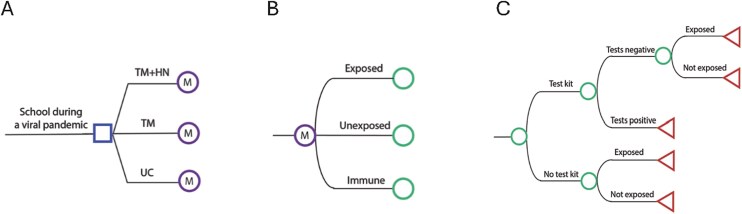
Decision analytic models. Blue square indicates decision nodes, purple circles indicate Markov nodes (with branches representing health states), green circles indicate chance nodes, and red triangles indicate terminal nodes. A) Depicts the interventions; B) depicts the health states; C) depicts the within-cycle probabilities of COVID-19 tests and exposures.

Analysis was conducted from 2023-2024 using TreeAge Pro 2024 R2 (TreeAge Software, LLC, Williamstown, MA). We constructed a decision analytic computer simulation model to compare the cost-effectiveness of the 3 arms of the study (UC, ITM, ITM + HN) using the school district perspective (Fig. 1). We analyzed the model for families of students and used a time horizon of an academic year (9 months). Because our time horizon was <1 year, we did not discount future outcomes. After entering the model, a family could be exposed to COVID-19.^24^ Families in the UC arm received unidirectional text messages every three weeks reminding them about the availability of testing. Families in the ITM and ITM + HN arms were then sent a monthly chain of bidirectional text messages offering test kits and following up to ask about the results, repeated for each month in the school year. Families cycled between exposed, unexposed, and immune to COVID-19. Immunity was assumed to last for 2 months.^25^

### Model Inputs

Input parameter values and their sources are shown in Table 1.

**Table 1:** Input parameters

Parameter	Value	Source
***Costs***		
Yearly salary of a health navigator (with benefits)	$55 000	SCALE UP Counts study
Test kit (includes 4 tests)	$39.76	SCALE UP Counts study
Shipping	$15.43	SCALE UP Counts study
Text message startup costs		
20 hours of program manager time	$1670	US BLS^31^
8 hours of software engineer time	$511	US BLS^32^
Server acquisition cost	$450	SCALE UP Counts study
Test server	$450	SCALE UP Counts study
Total	3431	
Yearly maintenance cost for the text message system		
Server maintenance	$350	SCALE UP Counts study
10 hours of data manager time	$554	US BLS^33^
10 hours of software engineer time	$639	US BLS^32^
Total	$1543	
Per text message cost	$0.0079	Twilio^30^
***Probabilities***		
Probability of initial exposure to COVID-19	0.41	Toth (2021)^28^
Opt out, ITM & ITM + HN*	0.93	SCALE UP Counts study
Opt out, UC	0.03	SCALE UP Counts study
Opt to pick up a test kit at school	0.39	SCALE UP Counts study
Opt to have a test kit mailed to you	0.61	SCALE UP Counts study
Opt to have a test kit mailed to you, UC	0.08	SCALE UP Counts study
Opt to pick up a test kit at school, UC	0.02	SCALE UP Counts study
Reporting testing occurred, ITM & ITM + HN	0.28	SCALE UP Counts study
Reporting testing occurred, no text messaging system	0.17	SCALE UP Counts study
Reporting testing occurred, after contact with health navigator	0.33	SCALE UP Counts study
Probability of a child testing positive for COVID-19	0.64	SCALE UP Counts study
Adult tested positive for COVID-19 after child tested positive	0.48	SCALE UP Counts study
Adult tested positive after child tested negative	0.28	SCALE UP Counts study
***Other***		
Number of families in the school district	8330	SCALE UP Counts study
Average number of adults per family	2	Fredde (2022)^34^
Average number of children per family	2	By assumption
Latency days for a test kit to be mailed to the family	2[0-5]	By assumption
Latency days for a test kit to be picked up at school	0[0-2]	By assumption
Tests per test kit	4	Program data
School days missed for a positive COVID-19 case	5[3-8]	CDC guidelines^29^
Workdays missed for a positive COVID-19 case	5[3-8]	CDC guidelines^29^

*Abbreviations:* US BLS, United States Bureau of Labor Statistics; CDC, United States Centers for Disease Control and Prevention; ITM, intensive text messaging; ITM+HN, intensive text messaging plus health navigation.

^*^Opt out, ITM & ITM + HN includes participants who did not respond to any messaging, including the option to opt out. These participants were included in the opt out parameter.

### Costs

The cost of sending a text message was taken from the Twilio (used by DHARE to deliver bidirectional text messages) website^26^ and applied to all three arms of the study. The school districts were assumed to already have a unidirectional messaging system in place, so the UC arm did not include any startup costs for the text message system. For the ITM and ITM + HN arms, the startup cost of the bidirectional text message system included 20 hours of a program manager, 8 hours of a software engineer, server acquisition costs, and a test server*.*^26-29^ Once the system was in place, yearly maintenance was required for data collection and upkeep of the program. This included the yearly license fee for the telecommunications component of DHARE responsible for development and delivery of intervention text messages, 10 hours of software engineer time for system updates, and 10 hours of a data manager’s time.^26^^,^^28^^,^^29^ The cost of a health navigator was based on the yearly salary estimate from the U.S Bureau of Labor Statistics for a similar position. All costs are presented in 2024 US dollars.

### Effectiveness Outcomes

The effectiveness outcomes were the number of COVID-19 tests taken, the number of missed school days avoided, and the number of missed workdays avoided. The number of missed work or school days avoided was the sum of the days missed waiting for the test to arrive and the number of days recommended for isolation for a positive COVID-19 case (5 days, range: 3-8) based on the CDC guidelines.^25^ The same range was used for the number of workdays missed due to a positive COVID-19 case. The number of latency days for the test kit was assumed to be 2 (range: 0-5) for a test kit that was delivered in the mail and 0 (range: 0-2) for a test kit picked up at school.

COVID-19 testing could affect missed school and workdays in two different ways: A negative test result could indicate that symptoms were not due to COVID-19 or that an individual did not need to isolate after an exposure, reducing the number of missed days. Or, a positive test result could indicate a need to isolate to prevent transmission, increasing missed days. For this study, we treated the default state as an individual isolating for the required length of time if they exhibited symptoms or were exposed to COVID-19, so missed school and workdays could only decrease with testing.

### Probabilities

The probabilities of a family opting into the program, requesting tests, reporting that testing occurred, and having a positive test came from the SCALE-UP Counts study.^30^ The probabilities of requesting a test, reporting testing, and test results only included individuals with valid responses or a text that corresponded to one of the options from the prompted questions. Individuals who did not respond to the prompt were considered non-responders and were only included in the initial likelihood of opting out. This use of only valid responses explains the high positive response rates for this study. The probability of all groups opting to pick up a test kit was based on information from one school in SCALE-UP Counts with the most complete data.

### Other Parameters

The size of the school district was taken from the average number of families enrolled in the program per school, excluding those without a valid phone number. We assumed that each family comprised of two adults and two children^31^ and that test kits contained 4 tests.

### Statistical Analysis

We conducted our analysis by running 8330 hypothetical families through our model for a 9-month time horizon. We assessed the relative value of different strategies by constructing an incremental cost-effectiveness ratio (ICER) by dividing the difference in cost by the difference in effectiveness between two strategies. We assessed the robustness of the model by running one-way sensitivity analyses in which each input parameter was varied by ranges available in the literature or by +/-20% if no ranges were available. In addition, we conducted probabilistic sensitivity analyses (PSA), in which each parameter was varied simultaneously across 10 000 Monte Carlo simulations. Cost, probability, and number of days missed parameters were assumed to follow gamma, beta, and Poisson distributions, respectively. We also ran a similar simulation in which costs varied by +/-75%.

### Alternative Scenarios

We analyzed our model under several alternative scenarios. In the first of these scenarios, we increased the percentages of COVID-19 testing and positive test results by 150% for individuals who were aware they had been exposed. In the second alternative analysis, we assumed that the school district did not have to purchase the COVID tests but were given them free of charge by a governmental agency. In the final alternative analyses, we varied the size of the school in the model: small (300 students), medium (550), and large (1000).^32^ For each school size, we ran the model once assuming one school-aged child per family and again assuming two school-aged children per family.

## RESULTS

### Base Case Scenarios

The results of the base-case analyses are shown in Table 2. UC was the least expensive strategy ($94 087 overall, $11 per family) followed by ITM ($239 719 overall, $29 per family) and ITM + HN ($284 604 overall, $34 per family). ITM + HN resulted in the most COVID-19 tests taken (9072) and the most missed school days avoided (8480), followed by ITM (5056 and 4700, respectively) and UC (2056 and 1790, respectively). When comparing the strategies to find the optimal one, the ITM strategy was eliminated from consideration because a mix of the two remaining strategies would yield more COVID-19 tests taken at a lower cost, a concept referred to as extended dominance. In other words, whether maximizing the number of tests taken or minimizing costs, ITM would never be chosen as the optimal strategy. The ICER for ITM + HN relative to UC was $27/COVID-19 test taken (Table 3). Results were similar in each of the alternative scenarios (Supplementary Tables S1 and S2).

**Table 2:** Cost and effectiveness results

	Costs		Effectiveness		
Strategy	Per family	Total for school district	COVID tests taken	Missed school days avoided	Missed workdays avoided
UC	$11	$94 087	2056	1790	2140
ITM	$29	$239 719	5056	4700	4920
ITM + HN	$34	$284 604	9072	8480	9320

*Abbreviations:* UC, usual care; ITM, intensive text messaging; ITM + HN, intensive text messaging plus health navigation.

**Table 3:** Incremental cost and effectiveness results

	Incremental costs		Incremental effectiveness			ICERs		
Strategy	Per family	Total for school district	COVID tests taken	Missed school days avoided	Missed workdays avoided	ICER ($/COVID test taken)	ICER ($/missed school day avoided)	ICER ($/missed workday avoided)
UC	–	–	–	–	–	–	–	–
ITM + HN	$23	$190 517	7016	6690	7180	$27.15	$28.48	$26.53

*Abbreviations:* UC, usual care; TM, text messaging; ITM + HN, intensive text messaging plus health navigation; ICER, incremental cost-effectiveness analysis. In cost-effectiveness analyses, a strategy is eliminated due to extended dominance if it has a higher cost and lower effectiveness than a combination of the other two strategies. This was the case here with ITM.

### Sensitivity Analyses

The parameters that most impacted costs were the cost of the COVID-19 test kit, cost of mailing the kit, the number of families in the school district, and the probability of initially opting out. The parameters that most affected the number of missed school days avoided (Supplementary Fig. S1) were the number of families in the school district, the probability of a child testing positive, the likelihood of opting for testing, the cost of the test, and the cost of mailing the test. The PSA results are shown as cost-effectiveness acceptability curves in Supplementary Fig. S2 for the COVID-19 tests taken effectiveness measure. Above a willingness-to-pay of $35 per test taken, the ITM + HN strategy had the highest probability of being cost effective compared to the other strategies.

## DISCUSSION

In this study, we conducted an economic evaluation of strategies designed to increase COVID-19 testing access for K-12 school students and their families. Results indicated that ITM + HN was only slightly more expensive than the ITM alone but yielded substantially more benefits in the form of more tests taken. ITM was more expensive and less effective than UC and ITM + HN. Relative to UC, the ITM + HN strategy yielded ICERs of $27/COVID test taken, $28/missed school day avoided, and $27/missed workday avoided. These results were most sensitive to the size of the school, the probability of opting out of the intervention, and the costs of the test kits.

The findings have several implications for school districts and policymakers. As schools face future pandemics or other disruptive events and consider strategies to create feasible and cost-effective digital interventions, decision makers should consider factors such as the length of time during which they will have access to funding for essential intervention components (e.g., COVID-19 test kits). While school district budgets are stretched increasingly thin, $284 000 represents a small fraction of many school districts’ annual budgets. For example, the Salt Lake City School District had an annual budget of $387 million in 2024-25. Within that budget, expenditures for student health and wellness programs can often be in the millions of dollars. For instance, state distributions for student health and counseling support in 2024 show multiple school districts in the state of Utah receiving over $1 million.^33^ In addition, a recent study reported that the annual operating budget of school-based health centers—which provide a wide range of physical and mental health screenings and services for school-aged children—was nearly $400 000 in 2024 US dollars.^34^ Taken together, these figures suggest that the cost of implementing ITM + HN would represent a modest share of typical school district spending on health-related programs. However, during a pandemic or other public health emergency, available resources for schools and school districts are often even more constrained. Our results provide concrete dollar estimates that can guide allocation of resources and preparedness planning for schools working in partnership with public health agencies under conditions of financial stress. In addition, our comparison of these costs with important school-related outcomes (i.e., an additional cost of $28 to avoid a missed school day) can help decision makers determine whether the increase in expenditures is worthwhile and scalable during future crises. Policymakers should also consider what funding is available for technical assistance for the development and maintenance of intervention infrastructures (e.g., text messaging system) if a school does not already have one in place. In considering the potential role for intervention supports such as a health navigator, schools may wish to build capacity for trained community health workers or existing staff who could help provide health navigation services to families during a disruptive event. Access to these services could allow schools to implement testing or other essential health services that could reduce missed school or work days, and thus minimize learning and economic losses.^14-17^

Several studies have examined the cost-effectiveness of different COVID-19 testing strategies in K-12 schools, along with clinical benefits and differences in disease transmission between strategies.^35^^,^^36^ While these studies have made valuable contributions, our study is unique and adds to these previous studies by evaluating interventions to improve the uptake of COVID-19 testing (rather than simply the testing strategies themselves) in K-12 schools. Because testing uptake is an important real-world obstacle, our findings may be of keen interest to school districts and policymakers. In addition, the parameter values for our model were generated from the prospective SCALE-UP Counts study so our findings should reflect real-world behaviors in the setting of an actual pandemic.

Our analysis had several limitations. First, as is the case with any simulation model, we made several simplifying assumptions. However, we ran many sensitivity and scenario analyses to describe how reliant our results were to these initial assumptions. Second, we used a static model rather than a dynamic one. In other words, there is no mechanism in our model that accounted for increased COVID-19 positivity rates if individuals with COVID do not test and continue to attend school. Future work could incorporate these feedback mechanisms through a compartmental or agent-based simulation model. Third, we assumed that the likelihood of a family unit being exposed to COVID-19 was the same value as the per person COVID-19 exposure rate. This was likely an underestimate, but since no value for the per family exposure rate was found for this time, the per person rate was used. Fourth, because our analysis used inputs from the SCALE-UP Counts study which was implemented in Utah, our results may not be generalizable to similar programs in other school settings. Finally, several of our input parameters were conditional on survey response, making them prone to non-response bias.

## CONCLUSIONS

In conclusion, in our economic evaluation conducted from the perspective of a school district, we found that ITM + HN increases the benefits of bidirectional text messaging compared to UC. However, this additional component also increases cost. Using a simulation model parameterized with data from the SCALE-UP Counts study and the published literature, the results from our study provide valuable information for education policy and decision makers to understand the tradeoffs when considering which screening and communication strategy would be optimal for a school population during a pandemic.

## List of Abbreviations

CDC, United States Centers for Disease Control and Prevention

DHARE, Digital Health to Advance Research Equity

COVID-19, coronavirus disease 2019

ICER, incremental cost-effectiveness ratio

ITM, intensive bidirectional text messaging

ITM + HN: intensive bidirectional text messaging plus health navigation

K-12, kindergarten through twelfth grade

PSA, Probabilistic sensitivity analyses

UC, usual care (unidirectional messaging)

US, United States

US BLS, United States Bureau of Labor Statistics

## Supplementary Material

Supplementary_materials_CLEAN_COPY_piaf111

## Data Availability

The data used in this study belong to the NIH Rapid Acceleration of Diagnostics- Underserved Populations (RADx-UP) program. De-identified data can be accessed through the NIH RADx Data Portal. Please contact the RADx-UP Coordination and Data Collection Center for more information.
