# Supplementary material for: Cost-effectiveness analysis of a text message system for COVID-19 testing for K-12 school communities
Source: J Pediatric Infect Dis Soc. 2026 Jan 22;15(1):piaf111. doi: 10.1093/jpids/piaf111 (PMC12825298; doi:10.1093/jpids/piaf111)
Supplement: Supplementary_materials_CLEAN_COPY_piaf111 [file supplementary_materials_clean_copy_piaf111.docx]

**Cost-Effectiveness Analysis of a Text Message System for COVID-19 Testing for K-12 School Communities:**

**Supplementary Material**

Description of SCALE-UP Counts study

Table S1

Table S2

Figure S1

Figure S2

**Description of SCALE-UP Counts study**

The interventions in the SCALE-UP Counts study included COVID-19 test kit distribution, text messages, and health navigation services and all were available in English and Spanish. The study was designed as a Sequential Multiple Assignment Randomized Trial, whereby participants were first randomized to UC versus ITM, and then ITM participants who were considered “non-responders” (i.e., no reply to text messages, including ones about testing outcomes) were stepped up to a second randomization of continued ITM versus ITM+HN.

Across all study arms, schools were provided access to at-home COVID-19 test kits for students,/parents and their household members free of charge. Test kits were provided at school administration offices and family engagement centers that provided services to school communities, or could be requested via text message (for ITM participants) or email (for UC participants) to be sent via overnight delivery to participants’ homes. Test use and outcomes were assessed via text messages sent to all participants every 23 days and other study assessments. This 23-day ‘intervention’ period was followed by 7 days during which outcomes of these text messages were assessed, resulting in a 30-day cycle that repeated throughout the duration of the study.

Text messages were implemented through a software platform (Digital Health to Advance Research Equity (DHARE)) which was developed at the University of Utah and has been used in multiple pragmatic clinical trials and studies.^24-27^ Participants in the UC group received one static, unidirectional text message every 3 weeks for up to 18 months that was consistent with public health messages about COVID-19. This included recommendations to test if exposed or experiencing symptoms, test pick up options, and how to email the study team for test kits. Participants in the ITM group were sent multiple cycles of bidirectional text messaging (number of cycles depended on the length of time a school was enrolled in the study) that aligned with the most current COVID-19 testing recommendations. The messages in each cycle: 1) assessed testing needs or interest, 2) provided guidance on testing based on participant responses and the option to request a test kit, and 3) provided an opportunity for participants to report completed testing. Follow-up text messages were sent out once per intervention cycle (23 days after intervention messaging concluded) and assessed testing outcomes, including number of household members tested and test results. Participants receiving ITM were deemed non-responders if they did not respond to these texts. These participants continued to receive ITM, but half were randomized to also receive a phone call from a health navigator who addressed testing barriers and provided information on testing access (ITM+HN).

**Table S1**: Additional scenario results

|  | **Costs** | | **Effectiveness** | | | **ICERs** | | |
| --- | --- | --- | --- | --- | --- | --- | --- | --- |
| **Strategy** | **Per family** | **Total for school districts** | **COVID tests taken** | **Missed school days avoided** | **Missed workdays avoided** | **$/COVID test taken** | **$/Missed school day avoided** | **$/Missed workday avoided** |
| *Alternative scenario 1*: Increased exposure ratio | | | | | | | | |
| UC | $13 | $113,462 | 2,596 | 1,820 | 2,730 | - | - | - |
| ITM | $29 | $245,974 | 5,960 | 3,330 | 6,660 | Excluded due to extended dominance | | |
| ITM+HN | $34 | $288,195 | 10,516 | 5,080 | 10,760 | 22 | 58 | 26 |
| *Alternative scenario 2*: Zero cost test kit | | | | | | | | |
| UC | $1 | $10,367 | 2,000 | 2,000 | 3,160 | - | - | - |
| ITM | $6 | $49,955 | 5,028 | 4,510 | 7,790 | Excluded due to extended dominance | | |
| ITM+HN | $12 | $103,281 | 9,216 | 8,570 | 13,740 | 11 | 12 | 8 |

*Note:* UC = usual care, ITM = intensive text messaging, ITM+HN = intensive text messaging plus health navigator, ICER = incremental cost-effectiveness analysis. A strategy (ITM, in this case) is eliminated due to extended dominance if it has a higher cost and lower effectiveness than a combination of the other two strategies.

**Table S2**: Cost per family of each missed school day avoided by school size

|  | **Costs** | | **Effectiveness** | | | **ICERs** | | |
| --- | --- | --- | --- | --- | --- | --- | --- | --- |
| **School Size** | **Per Family** | **Total for school district** | **Number of COVID tests** | **Missed school days avoided** | **Missed workdays avoided** | **ICER ($/COVID test)** | **ICER ($/Missed school day avoided)** | **ICER ($/Missed workdays avoided)** |
| *Small* | | | | | | | | |
| UC | $11 | $1,744 | 40 | 40 | 20 | - | - | - |
| ITM | $61 | $8,674 | 112 | 80 | 120 | Excluded due to extended dominance | | |
| ITM+HN | $426 | $63,660 | 172 | 230 | 200 | 387 | 417 | 377 |
| *Medium-small* | | | | | | | | |
| UC | $11 | $3,695 | 76 | 50 | 80 | - | - | - |
| ITM | $44 | $13,370 | 220 | 220 | 280 | Excluded due to extended dominance | | |
| ITM+HN | $226 | $67,862 | 376 | 320 | 300 | 206 | 231 | 204 |
| *Medium-large* | | | | | | | | |
| UC | $11 | $5,276 | 104 | 150 | 110 | - | - | - |
| ITM | $37 | $18,084 | 332 | 332 | 250 | Excluded due to extended dominance | | |
| ITM+HN | $146 | $72,179 | 568 | 460 | 490 | 132 | 143 | 27 |
| *Large* | | | | | | | | |
| UC | $11 | $11,867 | 284 | 240 | 230 | - | - | - |
| ITM | $33 | $32,455 | 600 | 550 | 670 | Excluded due to extended dominance | | |
| ITM+HN | $87 | $86,324 | 1072 | 990 | 1,190 | 80 | 85 | 75 |

*Note:* small school = 150 families, medium-small school = 300 families, medium-large school = 500 families, large school = 1,000 families, UC = usual care, ITM = intensive text messaging, ITM+HN = intensive text messaging plus health navigator, ICER = incremental cost-effectiveness analysis. A strategy (ITM, in this case) is eliminated due to dominance if it has a higher cost and lower effectiveness than a combination of the other two strategies extended dominance if it has a higher cost and lower effectiveness than a combination of the other two strategies.

**Figure S1.** Tornado diagram from the one-way sensitivity analysis for missed school days avoided


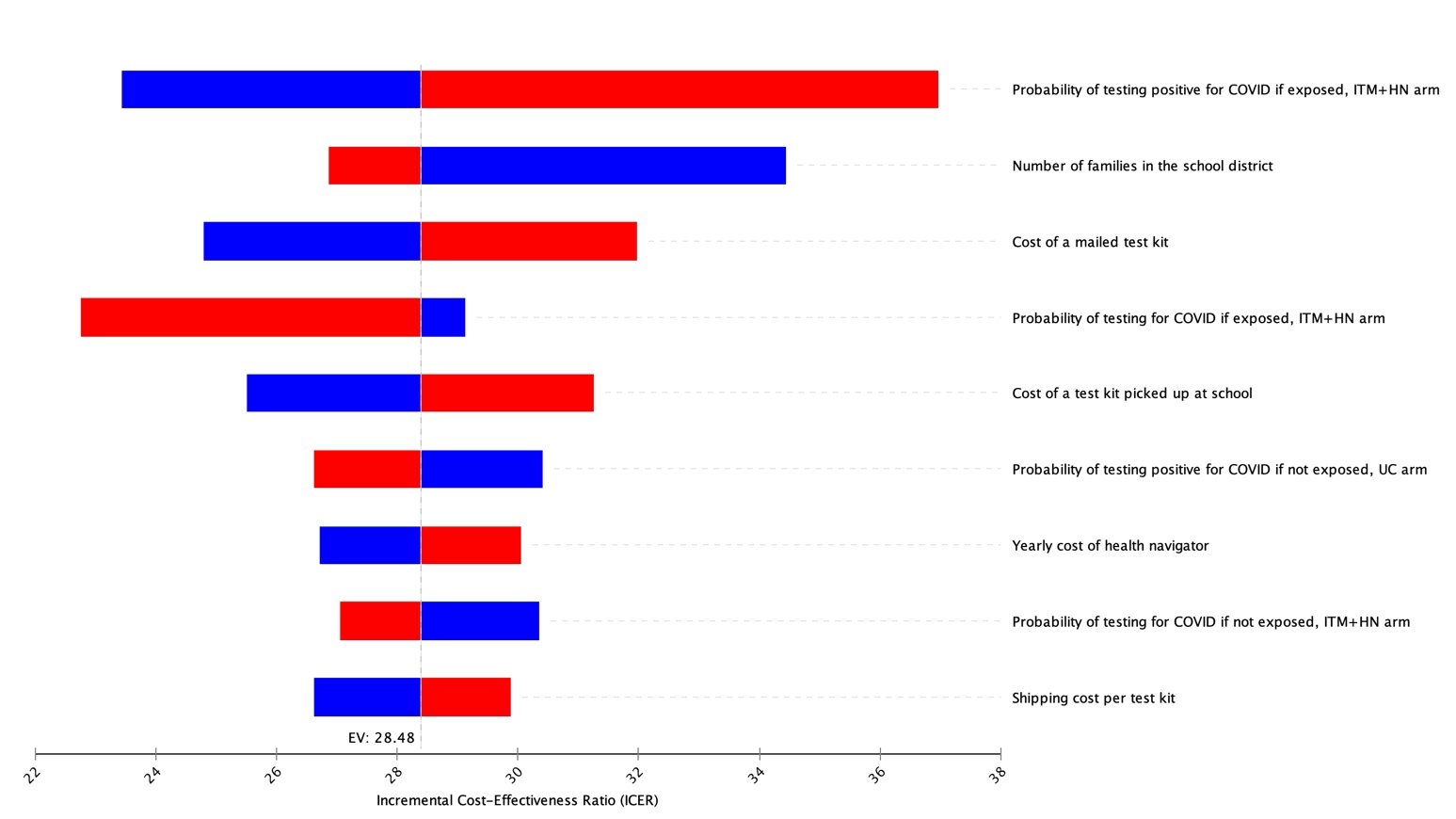


*Note:* Red and blue bars depict the size of the influence of the high and low end of the parameter value range on the incremental cost-effectiveness ratio (ICER), respectively. The vertical line at an ICER of 28.48 indicates the model result when each of the variables is set at their base-case value.

**Figure S2.** Cost-effectiveness acceptability curve for COVID-19 tests taken


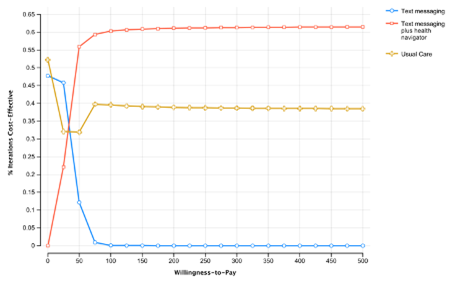


*Note:* Curves depicting the proportion of iterations from probabilistic sensitivity analysis in which each strategy was cost-effective (y-axis) across a range of willingness-to-pay thresholds (x-axis). When two curves cross, this indicates that there was an equal proportion of iterations in which those two strategies were deemed cost-effective at the corresponding willingness-to-pay threshold.
